# The association of radiologic body composition parameters with clinical outcomes in level-1 trauma patients

**DOI:** 10.1007/s00068-023-02252-6

**Published:** 2023-03-02

**Authors:** Arthur A. R. Sweet, Tim Kobes, Roderick M. Houwert, Rolf H. H. Groenwold, Pim Moeskops, Luke P. H. Leenen, Pim A. de Jong, Wouter B. Veldhuis, Mark C. P. M. van Baal

**Affiliations:** 1 Department of Surgery, University Medical Center Utrecht, PO Box 85500, 3508 GA Utrecht, The Netherlands; 2 Department of Radiology, University Medical Center Utrecht, Utrecht, The Netherlands; 3 Department of Clinical Epidemiology, Leiden University Medical Center, Leiden, The Netherlands; 4 Quantib, Rotterdam, The Netherlands

**Keywords:** Body composition, Computed tomography, Automatic segmentation, Trauma, Complications

## Abstract

**Purpose:**

The present study aims to assess whether CT-derived muscle mass, muscle density, and visceral fat mass are associated with in-hospital complications and clinical outcome in level-1 trauma patients.

**Methods:**

A retrospective cohort study was conducted on adult patients admitted to the University Medical Center Utrecht following a trauma between January 1 and December 31, 2017. Trauma patients aged 16 years or older without severe neurological injuries, who underwent a CT that included the abdomen within 7 days of admission, were included. An artificial intelligence (AI) algorithm was used to retrieve muscle areas to calculate the psoas muscle index and to retrieve psoas muscle radiation attenuation and visceral fat (VF) area from axial CT images. Multivariable logistic and linear regression analyses were performed to assess associations between body composition parameters and outcomes.

**Results:**

A total of 404 patients were included for analysis. The median age was 49 years (interquartile range [IQR] 30–64), and 66.6% were male. Severe comorbidities (ASA 3–4) were seen in 10.9%, and the median ISS was 9 (IQR 5–14). Psoas muscle index was not independently associated with complications, but it was associated with ICU admission (odds ratio [OR] 0.79, 95% confidence interval [CI] 0.65–0.95), and an unfavorable Glasgow Outcome Scale (GOS) score at discharge (OR 0.62, 95% CI 0.45–0.85). Psoas muscle radiation attenuation was independently associated with the development of any complication (OR 0.60, 95% CI 0.42–0.85), pneumonia (OR 0.63, 95% CI 0.41–0.96), and delirium (OR 0.49, 95% CI 0.28–0.87). VF was associated with developing a delirium (OR 1.95, 95% CI 1.12–3.41).

**Conclusion:**

In level-1 trauma patients without severe neurological injuries, automatically derived body composition parameters are able to independently predict an increased risk of specific complications and other poor outcomes.

**Supplementary Information:**

The online version contains supplementary material available at 10.1007/s00068-023-02252-6.

## Introduction

There has been an increasing interest in whether body composition affects outcomes in trauma patients in the past decade. As the utilization of computed tomography (CT) in the emergency department has substantially increased in the past years, there is a rising application of body composition assessment on these CT images that were previously taken for other clinical purposes [1–3]. Studies on body composition mainly focus on two body components: skeletal muscle mass and muscle quality to assess sarcopenia and fat mass to assess obesity [4, 5].

Sarcopenia, defined as a progressive and generalized skeletal muscle disorder associated with an increased likelihood of adverse outcomes, is regularly diagnosed by assessing the psoas muscle area only or the total muscle area at the cranio-caudal height of the third lumbar vertebra on axial CT [6, 7]. Sarcopenia, based on a low psoas muscle area, was associated with the in-hospital, 30-day, and 1-year mortality in trauma patients [4]. Several studies showed that a low psoas muscle density, as measured using the mean radiation attenuation in Hounsfield Units (HU) possibly as a marker of lower muscle quality and fatty degeneration, may also be a risk factor for poor outcomes in trauma patients [8, 9]. The effect of sarcopenia on complications in oncological populations has been investigated thoroughly, while only a few studies on this topic were performed in trauma populations [10].

Obesity is a metabolic disorder associated with cardiovascular disease and an increased morbidity and mortality rate [11]. Previous research focusing on the effects of obesity in trauma populations showed that obesity was associated with adverse in-hospital outcomes [12]. However, in these studies, obesity was defined by calculating the body mass index (BMI) [12]. As BMI is calculated using height and weight only, while not explicitly distinguishing between adipose tissue (compartment) and muscle mass, it could be a deceptive method to define obesity [13, 14]. Nowadays, numerous more accurate methods are described to assess fat mass, such as bioelectrical impedance analysis, dual-energy X-ray, magnetic resonance imaging, and CT [15–17]. A systematic review on quantification methods of fat mass underlined the clinical importance of visceral fat (VF) in contrast to subcutaneous fat (SF), as particularly VF has been associated with various diseases and in-hospital complications [18]. Studies on the effect of imaging-assessed visceral obesity on outcomes in oncological patients showed significant associations with a decreased survival [19]. Yet, studies on this topic in trauma patients remain scarce.

Even though studies have already shown associations between sarcopenia and poor outcomes in trauma patients, the effect of continuous body composition parameters, such as muscle mass and muscle density, on complications in trauma patients remains unknown [4]. The scarce literature on the effect of CT-measured fat mass on outcomes after trauma is even more ambiguous [20, 21]. Therefore, the present study aims to assess whether CT-measured muscle mass, muscle density, and visceral fat mass are associated with in-hospital complications and other clinical outcomes in level-1 trauma patients.

## Methods

### Study design and participants

A retrospective cohort study was conducted using information about all adult patients admitted to the University Medical Center Utrecht, a level-1 trauma center in the middle of the Netherlands, following a trauma between January 1 and December 31, 2017. Eligible patients were identified using procedural codes, and clinical data were obtained from the local trauma registry and the medical records. All trauma patients aged 16 years or older, who underwent a CT that included the abdomen within 7 days of admission, were included. Exclusion criteria were: severe neurological injury defined as an AIS-head ≥ 3, transfer to another hospital, administration of antibiotics or an active infection upon admission, and transfer from another hospital to our center after more than 24 h of admission. Neurologically injured patients were excluded as we hypothesized that the effect of this neurological injury would overshadow the effect of body composition on outcomes. Our institutional review board approved a waiver of consent.

### CT acquisition and evaluation

CT examinations were obtained with 16 × 0.75 mm collimation (Mx8000 IDT 16), 64 × 0.625 mm collimation (Brilliance 64, iQon Spectral), or 128 × 0.625 mm collimation (Brilliance iCT); all from Philips Medical Systems, Cleveland, OH, USA. Scans were primarily acquired at 120 kilovoltage peak (kVp) and in 3% of the patients at 100 or 140 kVp, with a range of 28–272 milliamperes per second depending on body size. According to our trauma protocol, all trauma patients that underwent CT immediately after admission received 2 mL/kg contrast medium using the split-bolus technique to assess the portal and arterial phase simultaneously. Axial images at the third lumbar vertebra (L3) level with a slice thickness of 0.9–5 mm were reconstructed and displayed with the intestine setting (window level: 30, window width: 400). Cross-sectional areas of muscles around the level of L3 have been shown to correlate well with whole-body muscle and fat mass [22, 23]. The L3 level was automatically identified and axial slices were automatically segmented by an artificial intelligence (AI) algorithm (Quantib Body Composition version 0.2.1, Quantib, Rotterdam, The Netherlands) [24]. This AI-driven segmentation method was also described in previous studies and showed high Dice coefficients compared to manual segmentation [25–27]. All segmented images were manually screened for errors (e.g., incorrect level L3 selection, or incorrect segmentation) and corrected if needed using the Medical Imaging Interaction Toolkit, a free, open-source software system.

### Explanatory variables

**Fig. 1:**
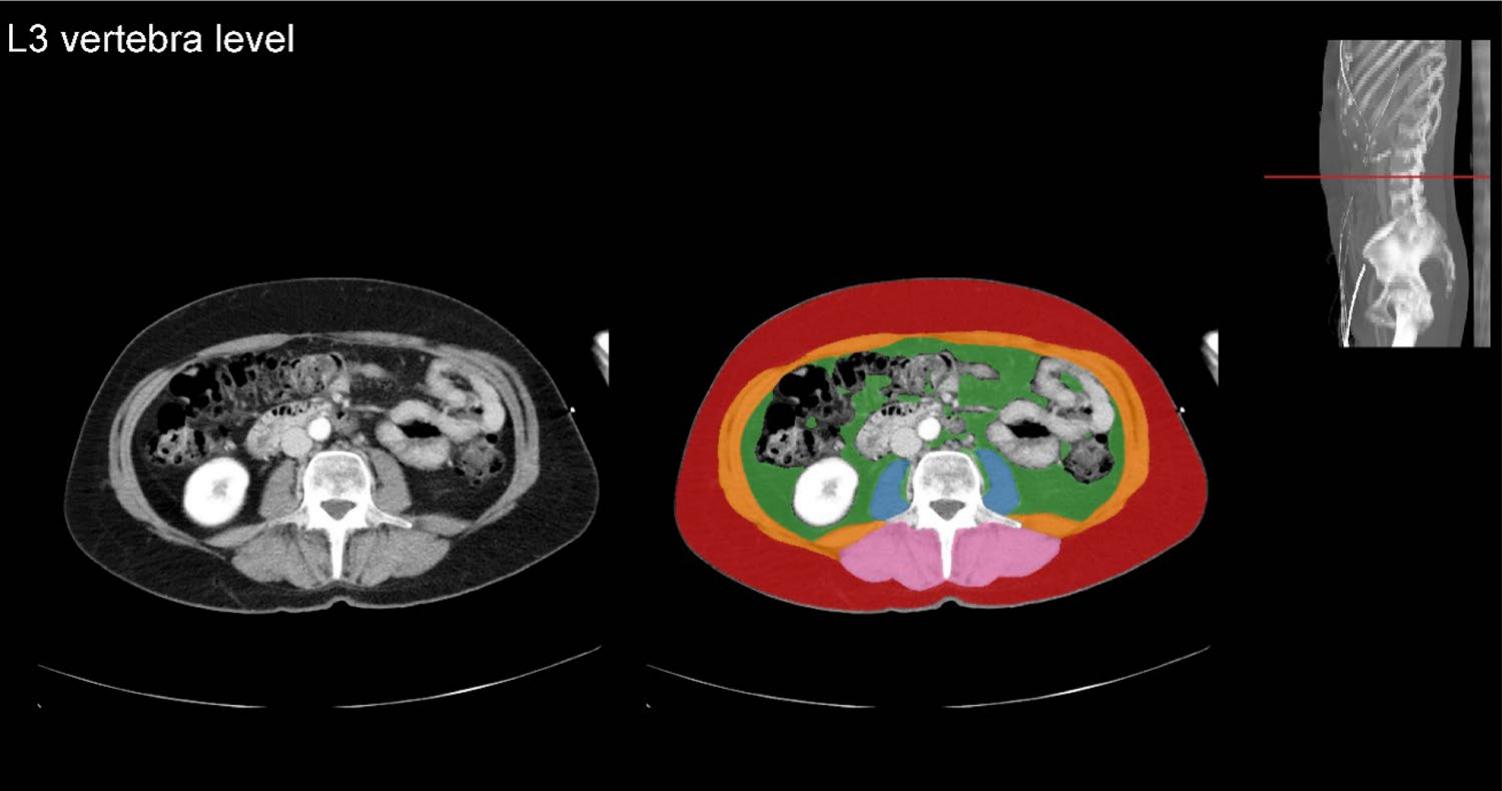
Segmentation of body compositions on an axial computed tomography image

Data on area and radiation attenuation of the following body components were automatically retrieved from the CT images: psoas muscles, abdominal wall muscles, para-spinal muscles, VF, and SF (Fig. 1). Body composition parameters were used for analysis only if the concerned body part was captured entirely on the axial CT slice and could therefore be adequately segmented. As the abdominal wall muscle area increased in patients with more VF and was not always fully visualized, we primarily used the psoas muscle to investigate associations with the outcomes and presented the association with total muscle area in the supplementary material. In the analysis of muscle areas, only the sub-areas with attenuation values above -30 HU within the segmented muscle areas were used to assure only pure muscle fibers were measured while excluding macroscopic muscular fat infiltrations. The psoas muscle index was calculated by dividing the psoas muscle area by the squared height of the patients (cm^2^/m^2^), which was used as a surrogate for muscle mass. Mean radiation attenuation values in HU of skeletal muscles were assessed based on the complete segmented muscle areas in which lower radiation attenuation represented more intramuscular fat deposition, indicating lower muscle fiber density. The effect of psoas muscle radiation attenuation was presented in the main tables, and the associations of total muscle radiation attenuation were added in the supplementary tables. As it was previously shown that predominantly VF, and not SF, was associated with complications, we focused on the effect of VF in this study. [18]

### Covariates

The following baseline characteristics were obtained from the local trauma registry: age, sex, American Society of Anesthesiologists (ASA) classification, mechanism of injury, maximum Abbreviated Injury Scale (AIS) for all body regions, Injury Severity Score (ISS), and Glasgow Coma Scale (GCS) score [28, 29]. ASA classifications were dichotomized into categories 1–2 and 3–4 to create categories with sufficient observations for analysis while still distinguishing between patients with moderate to severe comorbidities and patients with little or no comorbidities. Trauma patients were preferably scanned with both arms up to prevent creating artifacts in the abdominal CT images. However, one or both arms were kept down in immobile or severely injured patients. As the beam hardening created by the arms might decrease the measured radiation attenuation at the psoas muscle, all CT images were manually checked on this phenomenon to adjust for possible confounding appropriately.

### Outcomes measures

Primary outcome measures were all in-hospital complications, further specified into all infectious complications (i.e., pneumonia, urinary tract infections [UTI], wound infections, other infections), and delirium. Secondary outcome measures were hospital length of stay (HLOS), intensive care unit (ICU) admission, intensive care unit length of stay (ILOS) among patients admitted to the ICU, duration of mechanical ventilation (DMV) among patients who underwent mechanical ventilation, and unfavorable Glasgow Outcome Scale (GOS) score (i.e., severe disability, persistent vegetative state, or mortality).

### Statistical analysis

Baseline characteristics and outcome measures were presented as means ± standard deviation (SD) for parametric continuous variables, medians with interquartile range (IQR) for non-parametric continuous variables and ordinal variables, and numbers with proportions for categorical variables. In univariate analysis, the associations between skeletal muscle area or radiation attenuation or fat mass and outcome measures were analyzed using logistic regression analyses in binary outcomes and linear regression analyses in case of continuous outcomes. Subsequently, multivariable logistic and linear regression analyses were performed with adjustment for other factors (i.e., sex, age, ASA classification, ISS, and whether muscles were affected by beam hardening or not) to assess whether the body composition parameters were independently associated with the outcomes. The covariates added to the regression models were selected if they were likely to affect the outcomes based on clinical experience. Beam hardening was added to the model in the analysis of muscle radiation attenuation, as this could result from one or two arms down instead of up during CT, which was likely associated with poor outcomes. Significant associations of multivariable analysis were visually displayed using plots of the predicted absolute probability based on the multivariable regression models.

As data on patients’ height were missing in 61 cases (15.1%), a multiple imputation algorithm (Stata 13.0) was used to impute substitute data on height to properly estimate the psoas muscle index of these 61 patients to complete the dataset. Data on VF and total muscle area were missing in 23 (5.7%) and 33 patients (8.2%), respectively, as this was not always completely captured on axial CT, which was also imputed using multiple imputations. Twenty imputed datasets were created based on an imputation model that included all covariates that were also added to the final logistic or linear regression models and complications as the primary outcome measure. To assess whether imputation did not excessively affect the findings of this study, a sensitivity analysis was performed on the effect of the original un-imputed body composition parameters on the outcomes and presented in the supplementary material.

To enhance the interpretability of the odds ratios (ORs) and regression coefficients from the output of the analyses of muscle radiation attenuation and VF, the values were standardized by calculating *Z*-scores and subsequently analyzed and presented in this fashion. In that case, the OR represents the relative increase in odds when the muscle radiation attenuation or VF values increases by one standard deviation. We assumed that VF was linearly associated with the outcomes, in which we hypothesized that higher fat areas were associated with increased risks on adverse outcomes. However, to consider the possibility of non-linear associations between VF and the outcomes, we also assessed the effect of square-transformed VF values on the outcomes. Likelihood ratio tests showed that adding the square-transformed VF to the model, instead of the original values, did not significantly improve model fit, whereas the original VF values did significantly improve the model fit, thereby confirming our assumption of the linear association. To fulfill the assumptions for linear regression analyses, all continuous outcome measures were log-transformed before analysis, and the output values were transformed back to the multiplicative scale.

All statistical analyses were performed using Stata 13.0 (StataCorp LP, College Station, TX, USA). A *p*-value of < 0.05 was considered statistically significant.

## Results

### Baseline characteristics

**Fig. 2:**
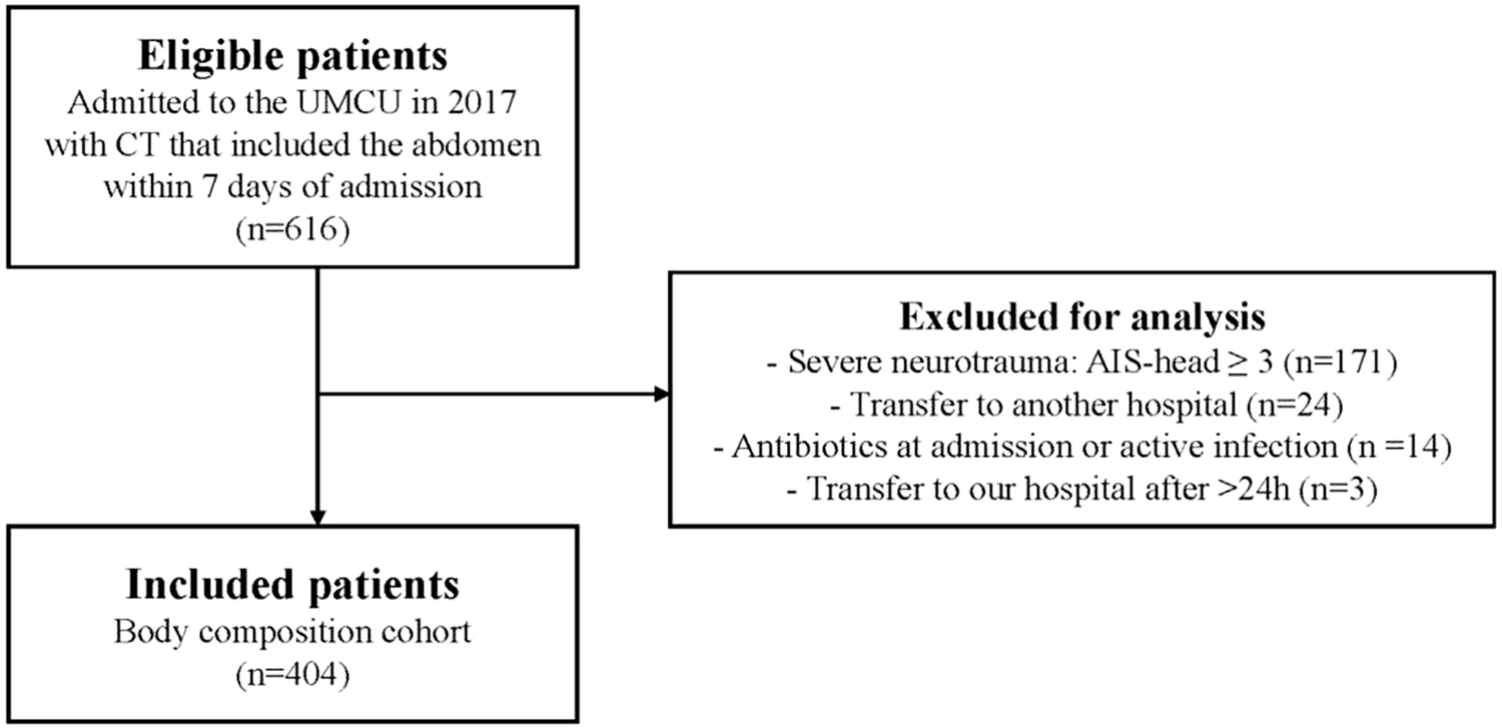
Flow diagram of the in- and exclusion process

A total of 616 trauma patients, who underwent a CT that included the abdomen within seven days of admission, were admitted to the University Medical Center Utrecht in 2017. After the exclusion process, 404 patients were included for analysis (Fig. 2). In 98% of all included patients, the abdominal CT was taken on the first or second day of hospital admission. In the included cohort, the median age was 49 years (IQR 30–64), and 66.6% were male (Table 1). Severe comorbidities (ASA 3–4) were seen in 10.9%, and the most prevalent mechanisms of injury in descending incidence were motor vehicle accidents, low- and high-energy falls, and bicycle accidents. The median ISS was 9 (IQR 5–14), and the median GCS score at admission was 15 (IQR 14–15). The mean psoas muscle index was 6.4 ± 2.0, and the mean psoas muscle radiation attenuation was 40.0 ± 15.8 HU. The median VF area of the complete cohort was 95.2 (IQR 43.4–162.0). Most patients (96.8%) were scanned at 120 kilo Volt and the mean milliampere-seconds was 112.1 ± 54.2.

**Table 1:** Baseline characteristics

**Variable**	Total cohort
	*n* = 404
Age at trauma, median (IQR)	49 (30–64)
Male, *n* (%)	269 (66.6)
Comorbidity ASA, *n* (%)	
1–2	360 (89.1)
3–4	44 (10.9)
Mechanism of injury, *n* (%)	
Motor vehicle accident	99 (24.5)
Motor cycle accident	47 (11.6)
Bicycle accident	62 (15.4)
Pedestrian	12 (3.0)
Low energy fall	88 (21.8)
High energy fall	64 (15.8)
Other	32 (7.9)
Maximum AIS-score, median (IQR)	
Head	1 (0–1)
Thorax	0 (0–3)
Abdomen	0 (0–0)
Spine	0 (0–2)
Lower extremities	0 (0–2)
Upper extremities	0 (0–1)
ISS, median (IQR)	9 (5–14)
ISS ≥ 16, *n* (%)	99 (24.5)
Glasgow Coma Scale score, median (IQR)	15 (14–15)
Psoas muscle area, mean cm^2^ ± SD	20.5 ± 7.4
PMI, mean cm^2^/m^2^ ± SD	6.4 ± 2.0
PMRA, mean HU ± SD	40.0 ± 15.8
VF, median (IQR)	95.2 (43.4–162.0)

*IQR* interquartile range, *SD* standard deviation, *ASA* American Society of Anesthesiologists, *AIS* abbreviated injury scale, *ISS* injury severity score, *PMI* psoas muscle index, *PMRA* psoas muscle radiation attenuation, *VF* visceral fat

### Outcome measures

Eighty-eight complications were seen in 17.3% of all patients, with the most prevalent complications being pneumonia (8.7%) and delirium (4.2%) (Table 2). In total, there was a median HLOS of five days (IQR 2–11), a median ILOS of three days (IQR 2–6), and patients who were ventilated had a median DMV of one day (IQR 1–3). At discharge, 24 (5.9%) patients had an unfavorable GOS score, indicating severe disability or mortality.

**Table 2:** Outcome measures

Variable	Total cohort
	*n* = 404
Complication, *n* (%)	70 (17.3)
Infectious complication, *n* (%)	61 (15.1)
Pneumonia, *n* (%)	35 (8.7)
Urinary tract infection, *n* (%)	13 (3.2)
Wound infection, *n* (%)	10 (2.5)
Other infectious complication, *n* (%)	13 (3.2)
Delirium, *n* (%)	17 (4.2)
Unfavorable GOS, *n* (%)	24 (5.9)
HLOS, median days (IQR)	5 (2–11)
ICU admission, *n* (%)	65 (16.1)
ILOS among patients on ICU, median days (IQR)	3 (2–6)
DMV among ventilated patients, median days (IQR)	1 (1–3)

*IQR* interquartile range, *ICU* intensive care unit, *GO*S Glasgow Outcome Scale, *HLOS* hospital length of stay, *ILOS* intensive care unit length of stay, *DMV* days on mechanical ventilation

### Skeletal muscle index

**Fig. 3:**
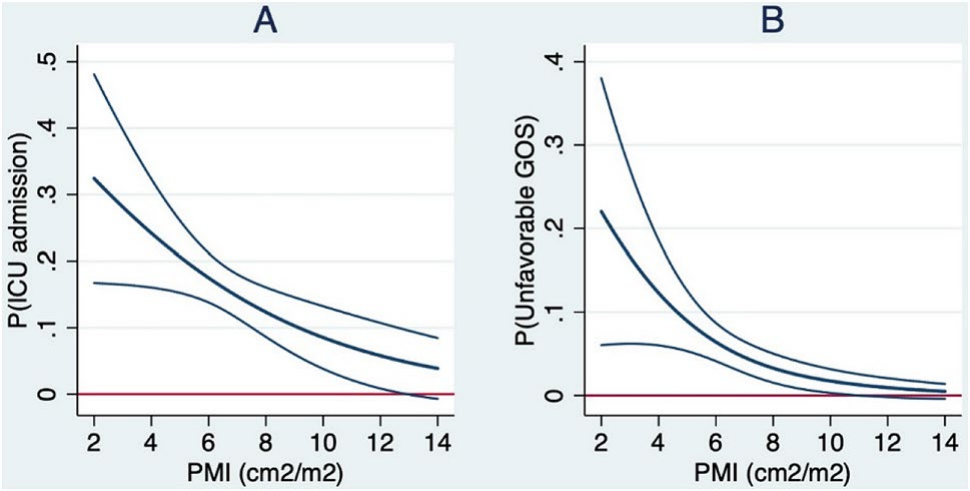
Plots of the absolute probability with 95% confidence interval of the outcomes (y-axis) predicted by the psoas muscle index (PMI) adjusted for covariates (x-axis). **A** Intensive Care Unit (ICU) admission, **B** unfavorable Glasgow Outcome Scale score (GOS)

Univariate logistic regression analysis showed that a higher psoas muscle index (i.e., more muscle mass) was associated with a lower risk of developing a complication (OR 0.87, 95% CI 0.76–0.99) (Table 3). A higher psoas muscle index was also associated with fewer UTIs and a shorter HLOS. Multivariable logistic regression analysis showed that after adjusting for covariates (i.e., age, sex, ASA, and ISS), the psoas muscle index was no longer significantly associated with the development of complications in general or any of the specific complications (Table 4). Multivariable analysis showed that for each unit increase in psoas muscle index, the odds of being admitted to the ICU decreased by 21% (OR 0.79, 95% CI 0.65–0.95), and the odds of being discharged with an unfavorable GOS decreased by 38% (OR 0.62, 95% CI 0.45–0.85) (Fig. 3).

**Table 3:** Univariate analysis of the effect of body composition parameters on outcomes

**Variable**	Psoas muscle index				Psoas muscle radiation attenuation				Visceral fat			
Logistic regression model	OR	95% CI	*t*-value	*p*-value	OR	95% CI	*z*-value	*p*-value	OR	95% CI	*t*-value	*p*-value
Complication	0.87	0.76–0.99	– 2.15	0.032	0.59	0.46–0.76	– 4.13	< 0.001	1.42	1.11–1.82	2.78	0.005
Infectious complication	0.91	0.79–1.04	– 1.42	0.15	0.65	0.51–0.85	– 3.20	0.001	1.37	1.06–1.77	2.38	0.017
Pneumonia	0.95	0.80–1.12	– 0.62	0.54	0.63	0.46–0.86	– 2.91	0.004	1.47	1.07–2.00	2.40	0.017
Urinary tract infection	0.67	0.49–0.91	– 2.56	0.010	0.82	0.49–1.38	– 0.76	0.45	0.85	0.45–1.60	– 0.51	0.61
Wound infection	0.87	0.63–1.18	– 0.90	0.37	0.67	0.40–1.15	– 1.46	0.15	1.40	0.81–2.43	1.20	0.23
Other infectious complication	1.21	0.94–1.56	1.48	0.14	0.84	0.49–1.43	– 0.65	0.52	1.64	1.02–2.61	2.06	0.039
Delirium	0.78	0.60–1.00	– 1.95	0.051	0.44	0.30–0.65	– 4.11	< 0.001	2.32	1.53–3.52	3.94	< 0.001
ICU admission	0.93	0.82–1.06	– 1.11	0.27	0.58	0.45–0.74	– 4.22	< 0.001	1.10	0.84–1.43	0.69	0.49
Unfavorable GOS	0.82	0.66–1.01	– 1.87	0.061	0.56	0.40–0.80	– 3.23	0.001	1.58	1.10–2.27	2.49	0.013

Linear regression model	β-coefficient	95% CI	*t*-value	*p*-value	β-coefficient	95% CI	*t*-value	*p*-value	β-coefficient	95% CI	*t*-value	*p*-value
HLOS	0.94	0.90–99	− 2.52	0.012	0.81	0.73–0.90	− 3.99	< 0.001	1.14	1.03–1.27	2.60	0.010
ILOS	1.02	0.90–1.15	0.27	0.79	0.86	0.71–1.51	− 1.57	0.122	1.14	0.90–1.44	1.10	0.28
DMV	0.99	0.89–1.10	− 0.18	0.86	0.84	0.72–0.99	− 2.12	0.038	1.21	0.99–1.47	1.95	0.056

*OR* odds ratio, *CI* confidence interval, *ICU* intensive care unit, *GOS* Glasgow Outcome Scale, *HLOS* hospital length of stay, *ILOS* intensive care unit length of stay, *DMV* days on mechanical ventilation

**Table 4:** Multivariable analysis of the effect of body composition parameters on outcomes with adjustment for other factors

Variable	Psoas muscle index				Psoas muscle radiation attenuation				Visceral fat			
Multivariable logistic regression model^a^	OR	95% CI	*t*-value	*p*-value	OR	95% CI	*z*-value	*p*-value	OR	95% CI	*t*-value	*p*-value
Complication	0.92	0.76–1.12	– 0.80	0.43	0.60	0.42–0.85	– 2.89	0.004	1.28	0.92–1.77	1.46	0.15
Infectious complication	0.95	0.78–1.17	– 0.45	0.65	0.65	0.46–0.94	– 2.32	0.020	1.30	0.92–1.83	1.47	0.14
Pneumonia	0.91	0.71–1.18	– 0.70	0.49	0.63	0.41–0.96	– 2.12	0.034	1.46	0.96–2.23	1.76	0.078
Urinary tract infection	0.91	0.60–1.38	– 0.46	0.65	0.86	0.38–.194	– 0.36	0.72	0.65	0.28–1.53	– 0.99	0.32
Wound infection	0.69	0.45–1.05	– 1.74	0.082	0.58	0.29–1.15	– 1.57	0.12	1.18	0.58–2.39	0.45	0.65
Other infectious complication	1.17	0.83–1.64	0.88	0.38	1.14	0.57–2.28	0.36	0.72	1.60	0.89–2.88	1.58	0.11
Delirium	0.92	0.63–1.33	– 0.44	0.66	0.49	0.28–0.87	– 2.45	0.014	1.95	1.12–3.41	2.36	0.018
ICU admission	0.79	0.65–0.95	– 2.53	0.011	0.54	0.38–0.77	– 3.41	0.001	1.19	0.85–1.66	1.02	0.31
Unfavorable GOS	0.62	0.45–0.85	– 2.96	0.003	0.57	0.35–0.92	– 2.30	0.021	1.44	0.90–2.29	1.52	0.13

Multivariable linear regression model^a^	β-coefficient	95% CI	*t*-value	*p*-value	β-coefficient	95% CI	*t*-value	*p*-value	β-coefficient	95% CI	*t*-value	*p*-value
HLOS	0.97	0.92–1.02	– 1.25	0.21	0.84	0.76–0.93	– 3.46	0.001	1.02	0.93–1.12	0.40	0.69
ILOS	0.94	0.80–1.11	– 0.70	0.49	0.80	0.65–0.99	– 2.10	0.040	1.07	0.81–1.43	0.50	0.62
DMV	0.89	0.79–1.01	– 1.78	0.80	0.77	0.66–0.90	– 3.30	0.002	1.06	0.86–1.31	0.60	0.55

*OR* odds ratio, *CI* confidence interval, *ICU* intensive care unit, *GOS* Glasgow Outcome Scale, *HLOS* hospital length of stay, *ILOS* intensive care unit length of stay, *DMV* days on mechanical ventilation

^a^Adjusted for age, sex, ASA, ISS, and beam hardening in case of radiation attenuation

Table 5 in the Supplementary Material shows that the total muscle index was not significantly associated with any of the outcomes. However, as total muscle area and VF were correlated (*ρ* = 0.28, *p* < 0.001), the hypothesized protective effect of muscle mass on the outcomes may have been neutralized by the negative effect of more VF.

### Skeletal muscle radiation attenuation

**Fig. 4:**
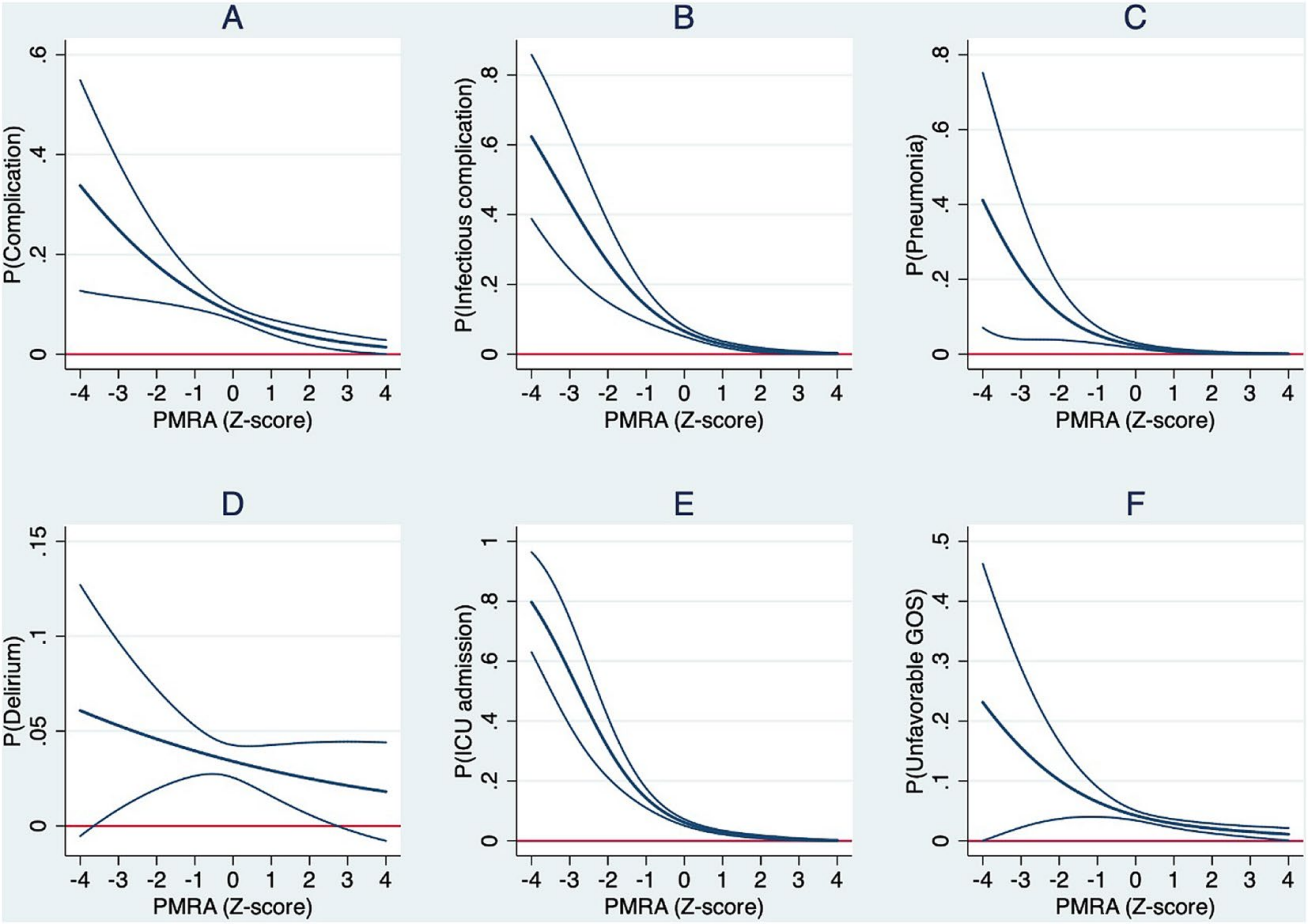
Plots of the absolute probability with 95% confidence interval of the outcomes (y-axis) predicted by standardized psoas muscle radiation attenuation (PMRA) (Z-score) adjusted for covariates (x-axis). **A** Complications, **B** infectious complications, **C** pneumonia, **D** delirium, **E** Intensive Care Unit (ICU) admission, **F** unfavorable Glasgow Outcome Scale (GOS) score

In univariate analysis, psoas muscle radiation attenuation was significantly associated with developing a complication and, more specifically, with the development of pneumonia and delirium (Table 3). Higher psoas muscle radiation attenuation, indicating higher muscle density, was also associated with a decreased risk of ICU admissions, decreased risk of unfavorable GOS score, and with shorter HLOS and DMV. After adjusting for covariates (i.e., age, sex, ASA, ISS, and beam hardening), higher psoas muscle radiation attenuation still predicted a decreased risk of developing complications (OR 0.60, 95% CI 0.42 – 0.85), pneumonia (OR 0.63, 95% CI 0.41–0.96), and delirium (0.49, 95% CI 0.28–0.87) (Table 4, Fig. 4). Higher psoas muscle radiation attenuation also remained associated with fewer ICU admissions (OR 0.54, 95% CI 0.38–0.77) and a decreased risk of an unfavorable GOS score (OR 0.57, 95% CI 0.35–0.92). Furthermore, one SD increase in psoas muscle radiation attenuation predicted a 16% decrease in HLOS (*p* = 0.001), 20% decrease in ILOS (*p* = 0.040), and a 23% decrease in DMV (*p* = 0.002). Total muscle radiation attenuation was not associated with the development of complications or any of the other outcomes (Supplementary Table 5).

### Visceral fat

**Fig. 5:**
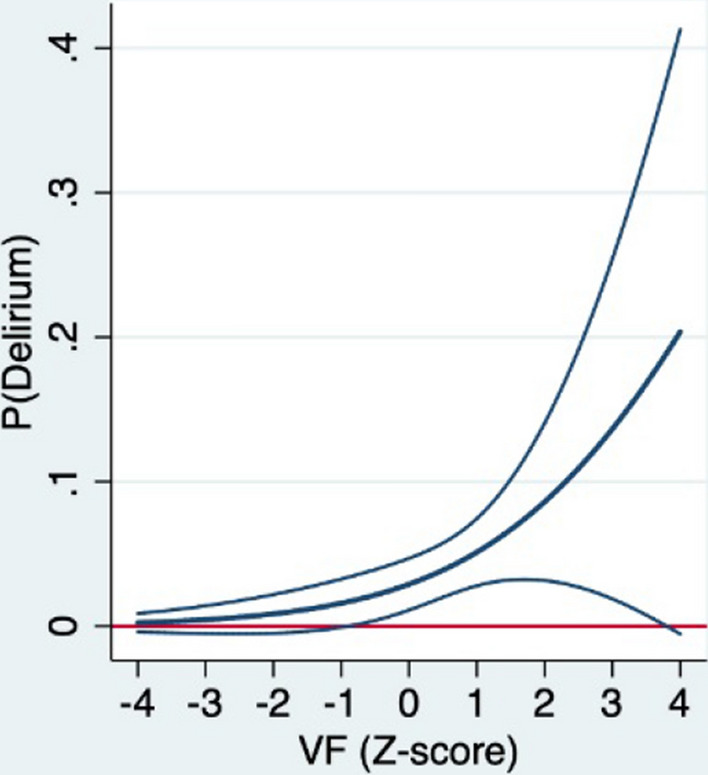
Plots of the absolute probability with 95% confidence interval of delirium (y-axis) predicted by standardized visceral fat area (*Z*-score) adjusted for covariates (x-axis)

Univariate logistic regression analysis showed that more VF was associated with more complications, infectious complications, pneumonia, other infectious complications, delirium, unfavorable GOS score, and longer HLOS (Table 3). After adjusting for other factors in multivariable logistic and linear regression analysis, only delirium was still significantly predicted by VF (Table 4). For each SD increase in VF, the odds of developing a delirium increased by 95% (OR 1.95, 95% CI 1.12–3.41) (Fig. 5). VF also appeared as a possible predictor of pneumonia (OR 1.46, 95% CI 0.96–2.23), yet without reaching statistical significance.

### Sensitivity analysis

In the supplementary material, the results of the sensitivity analysis with the original un-imputed body composition parameters were presented (Supplementary Tables 6 and 7). The results of multivariable regression analysis showed that imputation did not lead to any considerable changes.

## Discussion

This retrospective cohort study investigated the effects of muscle mass, muscle density, and visceral fat mass on complications and other clinical outcome parameters in level-1 trauma patients. A higher psoas muscle index—indicating more muscle—was independently associated with a decreased risk of ICU admission and a decreased risk of discharge with an unfavorable GOS score. Higher psoas muscle radiation attenuation—indicating less fatty muscle atrophy—predicted lower risk of developing a complication (i.e., pneumonia or delirium), a decreased ICU admission rate, fewer unfavorable GOS scores, and shorter HLOS, ILOS, and DMV. More visceral fat was independently associated with an increased risk of developing delirium.

Most studies on the effect of body composition on outcomes in trauma populations classified patients into sarcopenic or obese groups. Yet, continuous body composition parameters were used for analysis in this study. There were no significant associations between psoas muscle index and specific complications, which is in line with a systematic review of studies on sarcopenia among trauma patients by Xia et al. [4] Psoas muscle index appeared a possible predictor of wound infections, although not statistically significant (OR 0.69, CI 0.45–1.05). The only other study that specifically investigated this outcome showed that, instead of psoas muscle index, psoas muscle radiation attenuation predicted wound infections after adjusting for age and ASA (OR 1.12, 95% CI 1.03–1.22), which was not seen in our study [9]. We found that psoas muscle index was independently associated with an increased risk of an unfavorable GOS score, while the total muscle index was not associated with this outcome. One other study that investigated the effect of sarcopenia, based on the total muscle area, on GOS scores also described no significant associations, while another study did report a significant association in multivariable analysis [21, 30]. In the present study, no significant associations between HLOS and ILOS with the psoas muscle index were found, which is in line with the findings of most of the previous studies [4]. However, in a more recent study, an extremely low psoas muscle index was independently associated with a prolonged ILOS [31]. In contrast to our findings on psoas muscle index, lower psoas muscle radiation attenuation was associated with prolonged HLOS and ILOS, which is in accordance with previous studies [8, 9, 32]. Furthermore, two studies on the effect of psoas muscle radiation attenuation in trauma patients also reported significant associations with complications similar to our findings [8, 9]. Currently, it remains unclear to what extent muscle mass or muscle density and outcomes in trauma patients are causally associated. We hypothesize that muscle mass and density mainly act as frailty markers, predicting increased risks of adverse outcomes primarily caused by the entire frail physique of the patients. Also, it should be stressed that low psoas muscle area or density does not always necessarily correlate with sarcopenia. Isolated psoas atrophy occurs in the setting of spine surgery, lower back pain, degenerative lumbar instability, vertebral fracture, and deformity [33]. However, even though measuring the psoas muscle alone may be insufficient to assess generalized muscle loss in some cases, it can still be a predictor for poor outcomes, as shown in the present study. Similarly, even though low psoas muscle density does not necessarily correlate with overall fatty muscular atrophy, it served as a strong predictor in this study—even after adjusting for decreased density caused by beam hardening in patients scanned with their arms in the abdominal field of view. It seems plausible that the different included muscles follow distinct patterns in muscle loss or degeneration and may therefore also hold diverse predictive values for the outcomes.

Literature on the effect of CT-based adipose tissue on clinical outcomes in trauma patients is scarce. Docimo et al. showed more complications in trauma patients with a high visceral to subcutaneous fat ratio than in the control group (35.7% versus 13.3%) [20]. However, these groups were not comparable at baseline, and no adjustment for other factors was made. Poros et al. showed no significant effect of visceral and subcutaneous fat markers on DMV and ILOS in trauma patients [21]. They did show that obesity has some protective effects on the pre-hospital phase, as obese patients were less frequently intubated at the accident site and received less fluid during resuscitation before arriving at the emergency department, while not showing an increasing rate of shocks [21]. There are indeed studies reporting on the protective function of mild obesity in trauma patients, also known as the obesity paradox; however, no significant protective effects were found in our study population [34, 35].

To our knowledge, the present study is the most extensive study to investigate the effect of CT-derived visceral fat mass on complications in trauma patients. The findings of this study hold promising future implications in which artificial intelligence algorithms could aid in personalized medicine: CT scans are routinely acquired in a trauma setting and using AI quantitative assessment is more readily available than clinical scores, such ASA and AIS. In addition, scans acquired for any other medical purpose can also be used for opportunistic assessment of body composition to identify—and possibly even treat—patients at risk for certain complications. We found that visceral fat independently predicted an increased risk of delirium, which has not been described earlier. This finding adds to the current evidence on risks of the hormonally active form of body fat and further etiologic investigation is of interest and supported by our data. The purpose of this study, however, was primarily prognostication. Also related to prognostication, further evidence is needed before implementation. Ultimately, prediction models may indicate which patients are at highest risk of developing specific complications. In that case, our findings add to the development of such models.

This study has several limitations. First, the study population consisted of trauma patients in whom an abdominal or whole-body CT was indicated. Therefore, the results may not apply to all trauma patients admitted to the emergency department. Second, patients were included in case they underwent an abdominal CT in the first seven days of admission. As body composition may change during hospital admission, the body composition parameters might have been affected by this measurement delay. However, this effect should be limited, as in 98% of the patients the CTs were taken in the first two days of admission. Also, none of the 2% of patients scanned after the second day of admission were without food intake during the time before CT and the results were unchanged when excluding these 2% (data not shown). Third, because we excluded patients with severe neurological trauma—a strong independent predictor of worse outcome—the results of this study can only be used to identify the risk of complications in trauma patients without severe neurological injuries. However, these non-neurologically injured patients are the ones that could most benefit from the prediction of complications based on their body composition. Fourth, the numbers of events of certain complications were low in this cohort. Therefore, the regression models could have been over-fitted to our data, which might have resulted in some over- or underestimation of the associations between the body composition parameters and the outcomes. Last, mortality rates were not considered in the primary analysis, but these rates were low in this cohort (< 2%).

In conclusion, the present study showed that in level-1 trauma patients without severe neurological injuries, CT-derived body composition parameters are able to independently predict an increased risk of specific complications and other poor outcomes. Future research should further investigate the associations described in this study in other trauma populations. Also, the added predictive value of these body composition parameters, besides other proven outcome-specific predictors, should be further investigated, ultimately aiming to develop prediction models to assess personalized risks of developing complications.

## Availability of data and code

Data and code were stored in a secured Research Folder Structure of the UMC Utrecht and are available for future research.

## Supplementary Information

Below is the link to the electronic supplementary material.

Supplementary file1 (DOCX 23 KB)
