# Supplementary material for: The association of radiologic body composition parameters with clinical outcomes in level-1 trauma patients
Source: Eur J Trauma Emerg Surg. 2023 Mar 2;49(4):1947–58. doi: 10.1007/s00068-023-02252-6 (PMC10449658; doi:10.1007/s00068-023-02252-6)
Supplement: Supplementary file 1 — Supplementary file1 (DOCX 23 KB) [file 68_2023_2252_MOESM1_ESM.docx]

| **Table 5. Multivariable analysis the effect of total muscle index and density on outcomes with adjustment for other factors** | | | | | | | | | |
| --- | --- | --- | --- | --- | --- | --- | --- | --- | --- |
|  |  |  |  |  |  |  |  |  |  |
| **Variable** | Total muscle index | | | |  | Total muscle radiation attenuation | | | |
|  |  |  |  |  |  |  |  |  |  |
| *Multivariable logistic regression model** | *OR* | *95% CI* | *t-value* | *p-value* |  | *OR* | *95% CI* | *t-value* | *p-value* |
| Complication | 0.99 | 0.95 - 1.03 | -0.29 | 0.77 |  | 0.68 | 0.44 - 1.03 | -1.82 | 0.069 |
| Infectious complication | 1.01 | 0.97 - 1.06 | 0.66 | 0.51 |  | 0.77 | 0.49 - 1.20 | -1.16 | 0.25 |
| Pneumonia | 1.01 | 0.96 - 1.06 | 0.43 | 0.67 |  | 0.81 | 0.45 - 1.45 | -0.71 | 0.48 |
| Urinary tract infection | 0.95 | 0.85 - 1.05 | -1.02 | 0.31 |  | 0.57 | 0.27 - 1.18 | -1.51 | 0.13 |
| Wound infection | 0.98 | 0.89 - 1.07 | -0.53 | 0.60 |  | 0.62 | 0.26 - 1.52 | -1.04 | 0.30 |
| Other infectious complication | 1.05 | 0.98 - 1.12 | 1.38 | 0.17 |  | 1.58 | 0.65 - 3.89 | 1.00 | 0.32 |
| Delirium | 1.05 | 0.98 - 1.13 | 1.30 | 0.19 |  | 0.60 | 0.30 - 1.19 | -1.46 | 0.14 |
| ICU admission | 0.99 | 0.95 - 1.02 | -0.74 | 0.46 |  | 0.69 | 0.43 - 1.12 | -1.50 | 0.13 |
| Unfavorable GOS | 0.95 | 0.89 - 1.02 | -1.46 | 0.14 |  | 0.76 | 0.38 - 1.51 | -0.78 | 0.43 |
| *Multivariable linear regression model** | *β-coefficient* | *95% CI* | *t-value* | *p-value* |  | *β-coefficient* | *95% CI* | *t-value* | *p-value* |
| HLOS | 1.01 | 0.99 - 1.02 | 1.02 | 0.31 |  | 0.91 | 0.80 - 1.02 | -1.61 | 0.11 |
| ILOS | 1.00 | 0.97 - 1.04 | 0.12 | 0.91 |  | 0.80 | 0.56 - 1.14 | -1.26 | 0.21 |
| DMV | 0.99 | 0.97 - 1.02 | -0.39 | 0.70 |  | 0.77 | 0.58 - 1.00 | -1.98 | 0.054 |
| OR odds ratio; CI confidence interval; ICU intensive care unit; GOS Glasgow Outcome Scale; HLOS hospital length of stay; ILOS intensive care unit length of stay; DMV days on mechanical ventilation; *adjusted for age, sex, ASA, ISS | | | | | | | | | |
|  |  |  |  |  |  |  |  |  |  |

| **Table 6. Univariate analysis of the effect of original (unimputed) body composition parameters on outcomes** | | | | | | | | | |
| --- | --- | --- | --- | --- | --- | --- | --- | --- | --- |
|  |  |  |  |  |  |  |  |  |  |
| **Variable** | Psoas muscle index (n = 343) | | | |  | Visceral fat (n = 381) | | | |
|  |  |  |  |  |  |  |  |  |  |
| *Logistic regression model* | *OR* | *95% CI* | *z-value* | *p-value* |  | *OR* | *95% CI* | *z-value* | *p-value* |
| Complication | 0.88 | 0.77 - 1.01 | -1.78 | 0.074 |  | 1.43 | 1.12 - 1.83 | 2.85 | 0.004 |
| Infectious complication | 0.93 | 0.81 - 1.07 | -0.97 | 0.33 |  | 1.39 | 1.07 - 1.80 | 2.51 | 0.012 |
| Pneumonia | 0.97 | 0.81 - 1.16 | -0.29 | 0.77 |  | 1.48 | 1.08 - 2.01 | 2.45 | 0.014 |
| Urinary tract infection | 0.65 | 0.46 - 0.92 | -2.44 | 0.015 |  | 0.85 | 0.45 - 1.60 | -0.50 | 0.62 |
| Wound infection | 0.88 | 0.64 - 1.22 | -0.75 | 0.45 |  | 1.41 | 0.81 - 2.43 | 1.22 | 0.22 |
| Other infectious complication | 1.26 | 0.97 - 1.64 | 1.74 | 0.082 |  | 1.64 | 1.03 - 2.61 | 2.09 | 0.037 |
| Delirium | 0.74 | 0.55 - 0.99 | -2.01 | 0.045 |  | 2.33 | 1.54 - 3.54 | 3.98 | <0.001 |
| ICU admission | 0.95 | 0.83 - 1.09 | -0.68 | 0.50 |  | 1.12 | 0.86 - 1.45 | 0.83 | 0.41 |
| Unfavorable GOS | 0.81 | 0.64 - 1.02 | -1.76 | 0.078 |  | 1.59 | 1.11 - 2.27 | 2.53 | 0.011 |
| *Linear regression model* | *β-coefficient* | *95% CI* | *t-value* | *p-value* |  | *β-coefficient* | *95% CI* | *t-value* | *p-value* |
| HLOS | 0.96 | 0.91 - 1.01 | -1.49 | 0.14 |  | 1.14 | 1.03 - 1.27 | 2.56 | 0.011 |
| ILOS | 1.02 | 0.90 - 1.16 | 0.39 | 0.70 |  | 1.13 | 0.89 - 1.42 | 1.04 | 0.30 |
| DMV | 0.99 | 0.89 - 1.10 | -0.17 | 0.87 |  | 1.21 | 0.99 - 1.47 | 1.90 | 0.062 |
| OR odds ratio; CI confidence interval; ICU intensive care unit; GOS Glasgow Outcome Scale; HLOS hospital length of stay; ILOS intensive care unit length of stay; DMV days on mechanical ventilation | | | | | | | | | |
|  |  |  |  |  |  |  |  |  |  |

| **Table 7. Multivariable analysis of the effect of original (unimputed) body composition parameters on outcomes with adjustment for other factors** | | | | | | | | | |
| --- | --- | --- | --- | --- | --- | --- | --- | --- | --- |
|  |  |  |  |  |  |  |  |  |  |
| **Variable** | Psoas muscle index (n = 343) | | | |  | Visceral fat (n = 381) | | | |
|  |  |  |  |  |  |  |  |  |  |
| *Multivariable logistic regression model** | *OR* | *95% CI* | *z-value* | *p-value* |  | *OR* | *95% CI* | *z-value* | *p-value* |
| Complication | 0.93 | 0.75 - 1.14 | -0.74 | 0.46 |  | 1.28 | 0.92 - 1.77 | 1.47 | 0.14 |
| Infectious complication | 0.99 | 0.80 - 1.22 | -0.13 | 0.90 |  | 1.30 | 0.92 - 1.84 | 1.52 | 0.13 |
| Pneumonia | 0.95 | 0.73 - 1.22 | -0.41 | 0.68 |  | 1.46 | 0.96 - 2.22 | 1.76 | 0.078 |
| Urinary tract infection | 0.91 | 0.57 - 1.45 | -0.40 | 0.69 |  | 0.63 | 0.27 - 1.46 | -1.07 | 0.28 |
| Wound infection | 0.72 | 0.47 - 1.10 | -1.53 | 0.13 |  | 1.17 | 0.58 - 2.38 | 0.44 | 0.66 |
| Other infectious complication | 1.22 | 0.86 - 1.73 | 1.12 | 0.26 |  | 1.59 | 0.89 - 2.85 | 1.57 | 0.12 |
| Delirium | 0.86 | 0.57 - 1.30 | -0.71 | 0.48 |  | 1.90 | 1.10 - 3.29 | 2.30 | 0.022 |
| ICU admission | 0.80 | 0.66 - 0.97 | -2.28 | 0.023 |  | 1.19 | 0.85 - 1.66 | 1.02 | 0.31 |
| Unfavorable GOS | 0.61 | 0.44 - 0.85 | -2.88 | 0.004 |  | 1.43 | 0.90 - 2.29 | 1.51 | 0.13 |
| *Multivariable linear regression model* | *β-coefficient* | *95% CI* | *t-value* | *p-value* |  | *β-coefficient* | *95% CI* | *t-value* | *p-value* |
| HLOS | 0.98 | 0.93 - 1.47 | -0.58 | 0.56 |  | 1.02 | 0.93 - 1.13 | 0.46 | 0.65 |
| ILOS | 0.97 | 0.83 - 1.14 | -0.35 | 0.72 |  | 1.07 | 0.81 - 1.43 | 0.51 | 0.61 |
| DMV | 0.92 | 0.81 - 1.03 | -1.45 | 0.15 |  | 1.06 | 0.86 - 1.32 | 0.59 | 0.56 |
| OR odds ratio; CI confidence interval; ICU intensive care unit; GOS Glasgow Outcome Scale; HLOS hospital length of stay; ILOS intensive care unit length of stay; DMV days on mechanical ventilation, *adjusted for age, sex, ASA, ISS | | | | | | | | | |
|  |  |  |  |  |  |  |  |  |  |
